# Panniculitis in Children

**DOI:** 10.3390/dermatopathology8030037

**Published:** 2021-08-01

**Authors:** Isabelle Moulonguet, Sylvie Fraitag

**Affiliations:** 1 Cabinet de Dermatopathologie Mathurin Moreau, 75019 Paris, France; 2 Dermatology Department, Hôpital Saint Louis, 75010 Paris, France; 3 Dermatopathology Department, Hospital Necker Enfants-Malades, 75006 Paris, France; sylvie.fraitag@aphp.fr

**Keywords:** panniculitis, histopathology, autoinflammatory diseases

## Abstract

Panniculitides form a heterogenous group of inflammatory diseases that involve the subcutaneous adipose tissue. These disorders are rare in children and have many aetiologies. As in adults, the panniculitis can be the primary process in a systemic disorder or a secondary process that results from infection, trauma or exposure to medication. Some types of panniculitis are seen more commonly or exclusively in children, and several new entities have been described in recent years. Most types of panniculitis have the same clinical presentation (regardless of the aetiology), with tender, erythematous subcutaneous nodules. Although the patient’s age and the lesion site provide information, a histopathological assessment is sometimes required for a definitive diagnosis and classification of the disorder. In children, most panniculitides are lobular. At present, autoimmune inflammatory diseases and primary immunodeficiencies have been better characterised; panniculitis can be the presenting symptom in some of these settings. Unexplained panniculitis in a young child should prompt a detailed screen for monogenic immune disorders because the latter usually manifest themselves early in life. Here, we review forms of panniculitis that occur primarily in children, with a focus on newly described entities.

## 1. Introduction

Panniculitides comprise a heterogenous group of inflammatory diseases that involve the subcutaneous adipose tissue. In children, these disorders are rare but can be difficult to diagnose. In the present review, “adult” types of panniculitis that occasionally occur in children will only be cited or just briefly described. Certain subtypes of panniculitis (such as Rothmann-Makai syndrome, Weber-Christian disease, and cytophagic histiocytic panniculitis) are no longer considered to be specific entities. Furthermore, genetic lipodystrophies characterised by the loss of adipose tissue at some body sites will not be described here.

Most types of panniculitis have the same clinical appearance (regardless of the aetiology), with tender, erythematous subcutaneous nodules that occur mostly where fatty tissue is prominent (i.e., on the legs, thighs, buttocks, and cheeks) [1,2]. The histopathologic assessment of panniculitis is often difficult and requires adequate tissue samples—including subcutaneous fat. As non-specific histopathologic findings are common for late panniculitis lesions, it is necessary to sample early lesions [3]. With an appropriate biopsy specimen and an adequate clinical-pathological correlation, a specific diagnosis can be made in most cases of panniculitis [4].

Panniculitis are classified as lobular or septal, depending on where the inflammatory infiltrate is mainly located. However, the lobular or septal pattern is not related to the clinical findings; here, we will use the aetiological classification [4,5]. Most paediatric panniculitides have a lobular pattern (Table 1).

## 2. Specific Paediatric Panniculitides

### 2.1. Subcutaneous Fat Necrosis of the Newborn (SFN)

**Figure 1:**
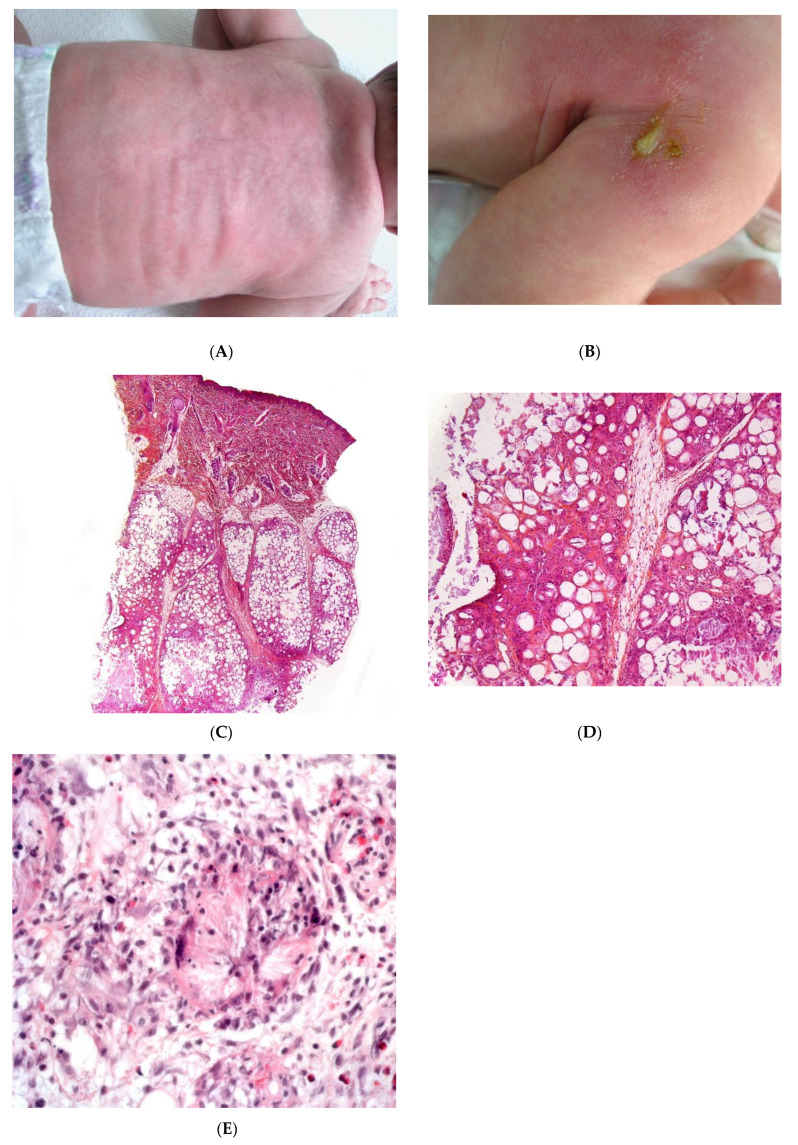
Subcutaneous fat necrosis of the newborn. (**A**) Indurated plaques on the neck and on the back of a newborn. (**B**) Significant purulent material of the shoulder *Photo Dermatology Department Hopital Necker Enfants Malades. Paris France.* (**C**) Lobular panniculitis without involvement of the dermis (original magnification ×20) (**D**) Many adipocytes are replaced by cells with finely eosinophilic granular cytoplasm that contain narrow needle-shaped clefts (original magnification ×100). (**E**) Needle-shaped crystals in radial fashion surrounded by histiocytes (original magnification ×400).

SFN is a rare variant of lobular panniculitis that appears in term or post-term newborns during the first days of life. The lesions are characterised clinically by indurated plaques and subcutaneous nodules that are primarily located on the buttocks, shoulders, cheeks, and thighs (Figure 1A). In some cases, the nodules and plaques can ulcerate and exude purulent material [6,7,8] (Figure 1B). The cause of SFN is unknown, although many cases are associated with perinatal asphyxia and meconium aspiration. The pathogenesis of SFN might be related to a greater saturated/unsaturated fatty acid ratio in the newborn, since saturated fatty acids have a greater tendency to crystallise in adipose tissue. SFN has an excellent prognosis, and spontaneous resolution within several weeks is common. However, delayed-onset hypercalcemia may occur, and so prolonged calcium monitoring is advisable. A biopsy is not always necessary because the clinical diagnosis is often straightforward. However, a biopsy can be useful in clinically misleading cases (such as multinodular cases, in particular) because the histological findings are specific.

#### 2.1.1. Histopathology

Most cases of SFN show characteristic features and are immediately recognisable as lobular panniculitis with necrosis of the fat lobule. It is a purely panniculitis, and the dermis is never affected. A high proportion of the adipocytes are replaced by cells with a finely eosinophilic, granular cytoplasm that contains radially arranged, narrow, needle-shaped clefts (Figure 1C–E). These clefts are characteristic of SFN and correspond to fatty acid crystals that have dissolved during sample processing. The adiponecrosis is associated with infiltration by inflammatory cells (lymphocytes, lipophages, and multinucleated giant cells), which can also contain these. Eosinophils and neutrophils can also be present. The neutrophil-rich variant of SFN can be difficult to distinguish from an infection [9]. Late-stage lesions can show septal fibrosis and areas of calcification within the fat lobule.

#### 2.1.2. Differential Diagnoses

Intracytoplasmic, needle-shaped fatty acid crystals are quite characteristic of SFN but can be also observed in post-steroid panniculitis and in “sclerema neonatorum” (SN). This very rare disorder manifests itself within a few days of birth in premature newborns, with comorbidities such as congenital heart disease and other major developmental defects [10]. From a histopathologic point of view, it is notorious that, despite striking clinical features, histopathology usually minimal changes. In the subcutaneous fat, the inflammatory infiltrate is sparse or even absent [2]. Given that (i) no new cases of SN have been reported in the last few years and (ii) SFN and SN have clinical and pathological similarities, they are now considered to be variants of the same disease. Hence, SN may be a severe form of SFN that primarily manifests itself in premature newborns [2].

### 2.2. Post-Steroid Panniculitis 

**Figure 2:**
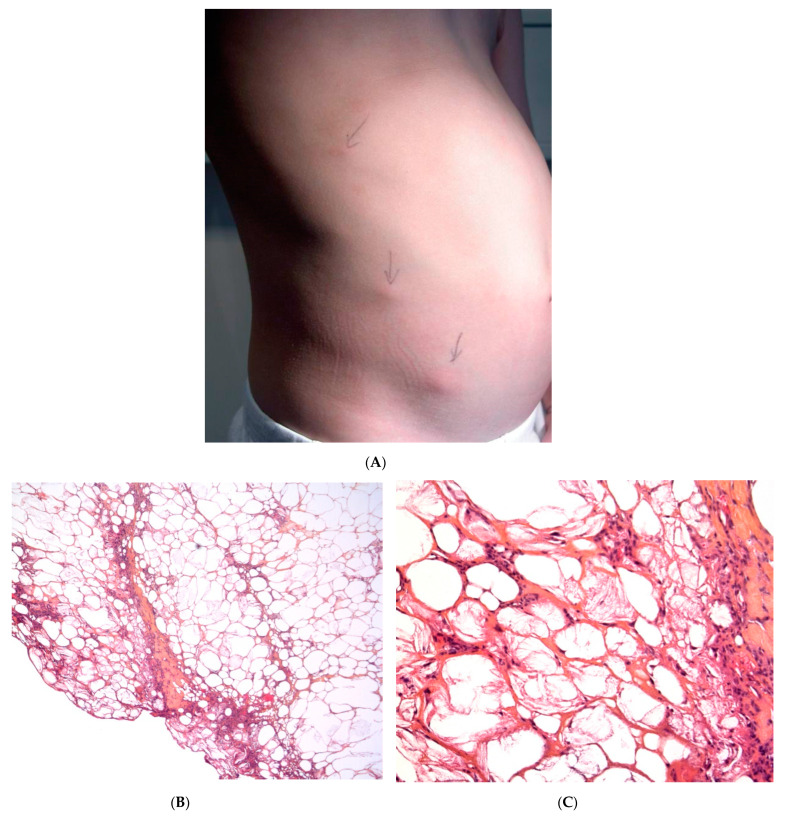
Post-steroid panniculitis. (**A**) Clinical presentation: subcutaneous nodules with overlying erythema. (**B**) Histopathologic features of post-steroid panniculitis: mostly lobular panniculitis with an inflammatory infiltrate of foamy histiocytes involving the fat lobules (original magnification ×40). (**C**) Needle-shaped clefts (original magnification ×400).

Post-steroid panniculitis is a very rare form of lobular panniculitis that has only been observed in children in whom systemic treatment with high-dose corticosteroids has been suddenly withdrawn. One to ten days after this withdrawal, erythematous subcutaneous nodules measuring 0.5 to 4 cm appear on the cheeks, the arms, or trunk (Figure 2A). The nodules may ulcerate, with scarring. The sudden withdrawal of corticosteroids might cause an increase in the saturated:unsaturated fatty acid ratio, and thus, crystal formation.

#### Histopathology

The findings are similar to SFN: a mostly lobular panniculitis features an inflammatory infiltrate of foamy histiocytes, with lymphocytes in the fat lobules. Some of the histiocytes show needle-shaped clefts in the cytoplasm (Figure 2B,C). In most cases, the inflammation is less intense and the crystals are less numerous than in SFN [11].

### 2.3. Cold Panniculitis 

Cold panniculitis is related to cold exposure. It is more frequent in infants and children than in adults—probably because of age-related differences in fat composition. Cold panniculitis usually affects the cheeks and chin and appear 48 to 72 h after cold exposure. In particular, cold panniculitis is reported in infants after the application of ice bags for the treatment of supraventricular tachycardia [12] and as “popsicle panniculitis” in children sucking ice cubes or popsicles.

The clinic features include firm, indurated erythematous nodules or plaques with ill-defined margins on the cheeks and the submental region. The prognosis is excellent, and the disorder resolves spontaneously over a period of weeks to months. A biopsy is not usually required if the history of a cold exposure is reported. However, distinguishing clearly between SFN and cold panniculitis is difficult in some cases [2]. Given that SFN requires prolonged calcium monitoring, a biopsy may be needed if direct exposure to cold has not been reported.

#### Histopathology

Cold panniculitis is a mostly lobular form of panniculitis. The infiltrate mostly comprises lymphocytes and histiocytes in the fat lobules. The inflammation can affect both the interlobular septa and lobules, and is most intense at the dermal-subcutaneous junction. The dermis usually shows both superficial and deep perivascular infiltrates composed mostly of lymphocytes, in the absence of vasculitis.

### 2.4. Autoinflammatory Diseases

**Figure 3:**
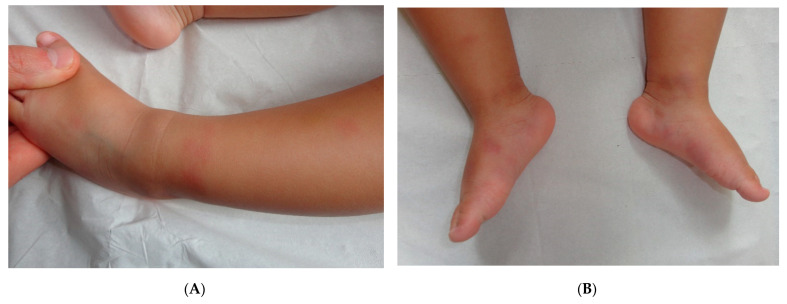

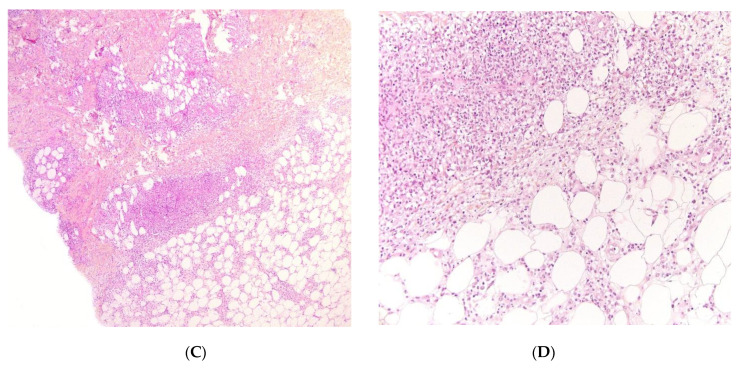
Panniculitis associated with inherited immunodeficiency. (**A**,**B**) Clinical presentation: Erythematous nodules of the leg of a 20 month-old boy *Photo Dermatology Department Hopital Necker Enfants Malades. Paris France* (**C**,**D**) Histopathology: inflammation of the deep dermis and the subcutis localised in the septa and the lobules composed of mixed infiltrate. (**C**) Original magnification ×80, (**D**) original magnification ×200.

**Figure 4:**
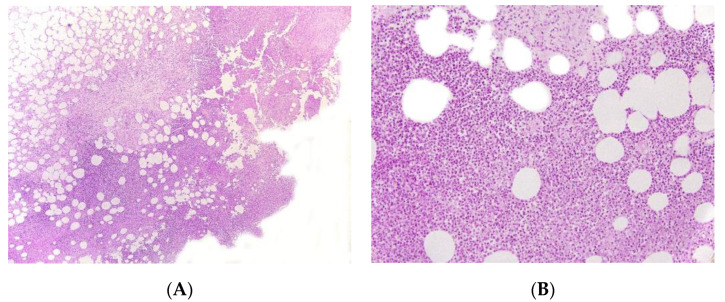
Panniculitis associated with inherited immunodeficiency. (**A**) Lobular panniculitis with dense neutrophilic infiltrate replacing part of the subcutis (original magnification ×80). (**B**) Closer view of showing entrapped adipocytes within the neutrophilic infiltrate (original magnification ×200).

Early-onset panniculitis with systemic inflammation had been reported in cases of autoinflammatory diseases with or without an associated immunodeficiency [13,14]. Most cases have variable, non-specific histopathologic features: lobular or septal panniculitis with a mixture of cells or with a predominance of neutrophils or lymphocytes (Figure 3 and Figure 4). However, the histopathologic features can sometimes have diagnostic value, such as the granulomatous infiltrate characteristic of Blau syndrome (BS) or the polyarteritis-nodosa-like vasculitis in deficiency of adenosine deaminase 2 (DADA2). The presence of early-onset panniculitis with systemic inflammation should prompt the physician to screen for autoinflammatory disorders.

#### 2.4.1. Autoinflammatory Diseases in the Absence of Immunodeficiency

##### Proteasome-Associated Autoinflammatory Syndromes (PRAAS)

PRAAS (OMIM 256040) are a group of distinct clinical entities that have recently been recognised to share a common molecular cause. They include joint contractures, muscle atrophy, microcytic anemia and panniculitis-induced lipodystrophy syndrome (JMP), Nakajo-Nishimura syndrome (NNS, also referred to as Japanese autoinflammatory syndrome with lipodystrophy, JASL), and chronic atypical neutrophilic dermatosis with lipodystrophy and elevated temperature syndrome (CANDLE). All these syndromes are characterised by the early onset of nodular, pernio-like, violaceous skin lesions with atypical neutrophil infiltrates, muscle atrophy, lipodystrophy, failure to thrive, and deformities of the hands and feet due to joint contractures. Recurrent periodic fever episodes and elevated acute phase reactant levels are usually present [15,16,17].

###### Histopathology

CANDLE is not a true panniculitis, since the reticular dermis and the hypodermis are affected and contain a perivascular and interstitial mononuclear inflammatory infiltrate. Many of the mononuclear cells exhibit large, vesicular, irregularly shaped nuclei, and thus, resemble atypical myeloid cells. Scattered mature neutrophils, some mature lymphocytes, and (in some cases) eosinophils are also present. Although leukocytoclasis is often present, there is no vasculitis [18]. Immunohistological studies have indicated that the cutaneous inflammatory infiltrate in CANDLE syndrome is polymorphous; it includes a mixture of immature myeloid cells that are strongly positive for myeloperoxidase, macrophages that are strongly positive for CD68 and CD163, and a moderate number of CD123-positive plasmacytoid dendritic cells.

##### Familial Mediterranean Fever

Familial Mediterranean fever classically consists of short, recurrent episodes of fever, serositis, arthritis, and erysipelas-like-erythema. The disorder results from mutations in the gene coding for pyrin (also known as marenostrin). Lobular panniculitis has been reported in adults but also in children, where tender, erythematous, bruise-like, warm, irregularly shaped nodules on the limbs and face coincide with the episodes of fever. The skin lesions heal as greyish macules without lipoatrophy. Panniculitis may be the main clinical manifestation, along with periods of fever [19]. 

###### Histopathology

Familial Mediterranean fever is a predominantly lobular neutrophilic panniculitis. Neither necrosis nor vasculitis is present [20].

##### Otulipenia

Otulipenia (also known as OTULIN-related autoinflammatory syndrome) is an autosomal recessive autoinflammatory disease caused by mutations in the *FAM105B* gene coding for OTU deubiquitinase with linear linkage specificity (OTULIN, a Met-1-specific deubiquitinase that downregulates the NF-kB signalling pathway). In clinical terms, patients present with early-onset, prolonged, recurrent episodes of fever, joint pain, abdominal pain, diarrhoea, and lymphadenopathy. A painful erythematous rash with nodules first noted in the neonatal period is the most frequent cutaneous manifestation.

###### Histopathology

Otulipenia is predominantly a septal form panniculitis with occasional vasculitis of small and medium-sized blood vessels [21].

##### BS

BS (also known as paediatric granulomatous arthritis) is usually caused by inherited dominant mutations in the *NOD2/CARD15* gene. The disorder can also present sporadically as “early-onset sarcoidosis” after an acquired gene mutation. The skin, eyes, and joints are commonly involved, although some BS patients do not exhibit the full clinical triad. Skin involvement is most prominent and typically appears before joint symptoms and then eye involvement. Together with a histological analysis, a detailed clinical exploration of skin lesions by an expert dermatologist may enable the diagnosis of this orphan disease in early childhood. Early-onset skin lesions have a homogeneous, stereotypical clinical presentation as non-confluent erythematous or pigmented millimetre-size papules. In contrast, late-stage skin lesions have a more heterogeneous clinical presentation and may be wrongly diagnosed as erythema nodosum (EN), ichthyosiform dermatosis, livedoid lesions, or vasculitis [22]. Vouters et al. reported four children with infantile onset lobular panniculitis, high fever, uveitis, arthritis, and systemic granulomatous inflammation without CARD15 mutation [23].

###### Histopathology

BS is a granulomatous panniculitis, with an inflammatory infiltrate of typical, non-necrotising, non-caseating “sarcoid type” epithelioid and multinucleated giant cell granulomas.

##### Tumour Necrosis Factor Receptor-Associated Periodic Syndrome (TRAPS)

TRAPS is the most frequent autosomal dominant autoinflammatory disease. Mutations in the *TNFRSF1A* gene (coding for the tumour necrosis factor receptor 1) induce the overproduction of interleukin-1b. In children, variants of TRAPS usually occur as recurrent, irregular febrile episodes with generalised myalgia, joint pain, abdominal pain, ocular lesions, and (in about 80% of cases) skin involvement. The most frequent skin lesions are painful, migratory, centrifugal, tender, non-purpuric, well-demarcated erythematous plaques. Other manifestations include urticaria-like plaques, generalised serpiginous plaques, and small-vessel vasculitis [24,25].

###### Histopathology

TRAPS is mostly a lymphocytic, lobular form of panniculitis.

#### 2.4.2. Autoinflammatory Syndromes with Inherited Immunodeficiency 

**Figure 5:**
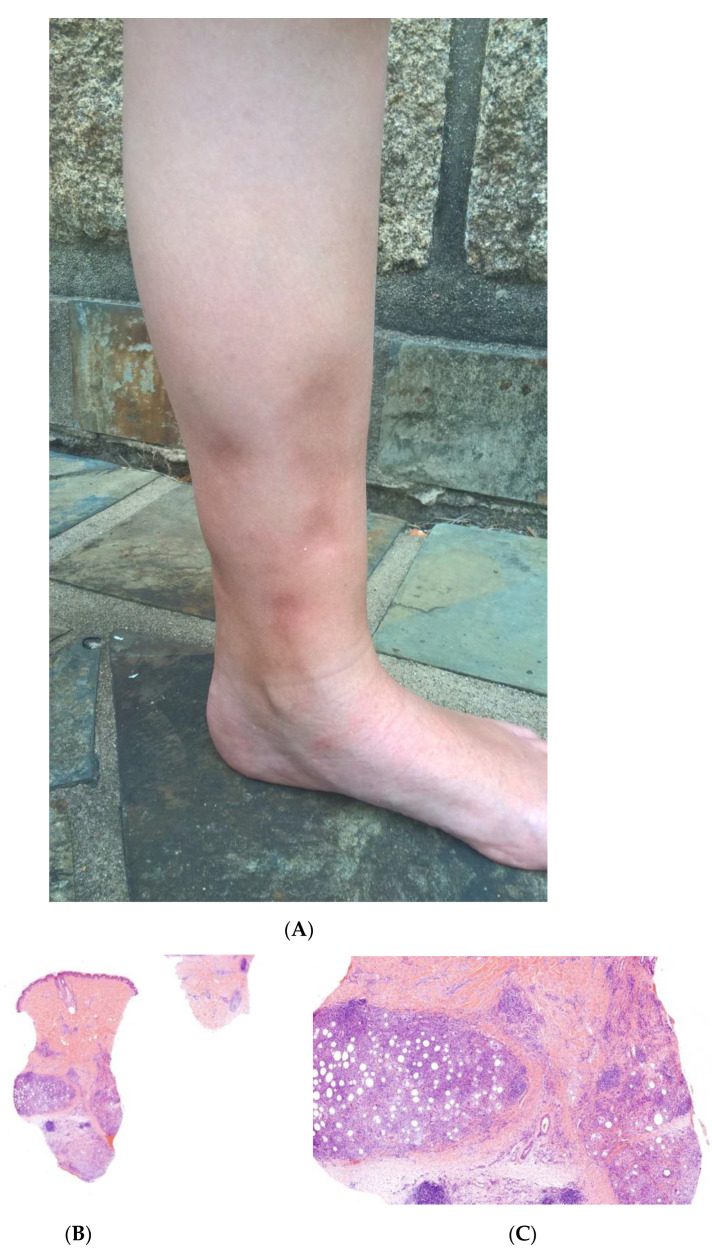
A Recurrent lipoatrophic panniculitis of children. (**A**) Areas of lipoatrophy in the leg *Photo Dermatology Department Hopital Necker Enfants Malades. Paris France*. (**B**,**C**) Lobular panniculitis with a mixed infiltrate, including neutrophils, as well as lymphocytes and macrophages. (**B**) Original magnification ×10, (**C**) original magnification ×40. *Photo Courtesy of Christina Mittledorf Dermatologie, Venerologie und Allergologie Göttingen Germany.*

**Figure 6:**
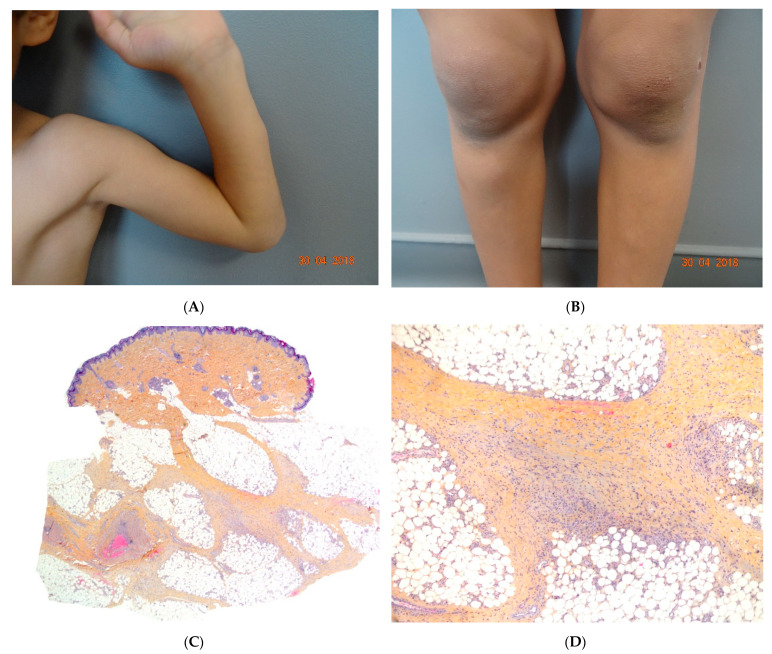
(**A**,**B**) Self-healing juvenile cutaneous mucinosis. Painful nodules of the limbs. *Photo Courtesy of Christine Bodemer Dermatology Department Hopital Necker Enfants Malades. Paris France*. (**C**,**D**) Chronic lobular panniculitis with features of proliferative fasciitis. (**C**) Original magnification ×20, (**D**) original magnification ×100.

Early-onset childhood panniculitis may reveal inherited immunodeficiency, and patients who present with unexplained panniculitis must undergo a detailed immunological screen because the clinical manifestations of immunodeficiency may not yet have emerged. An association with aseptic panniculitis was initially reported in infants with inherited immunodeficiency caused by mutations in the *GATA2* gene (coding for a zinc finger transcription factor) or *ADA2*. Other mutations (in *TRNT1*, *NFKB2*, and *LCK*) have since been reported [13] (Figure 4, Figure 5 and Figure 6).

##### Panniculitis in *GATA2* Deficiency

The transcription factor *GATA2* regulates haematopoietic differentiation, lymphatic development, and vascular development. Nearly 100 different *GATA2* mutations have been described. Germline mutations arise spontaneously but are then transmitted via autosomal dominant inheritance. Patients with *GATA2* mutations have very heterogeneous clinical presentations. The level of severity ranges from asymptomatic disease to life-threatening infections with respiratory failure and leukaemia. Up to 70% of patients with *GATA2* mutations have dermatological features (mainly genital or extragenital warts) and up to a third have EN or panniculitis (usually on the lower limbs). These conditions can have several causes (nontuberculous mycobacterial infections, bacterial infections, or autoimmune phenomena) and may constitute the first manifestation of disease [26,27].

###### Histopathology

Panniculitis in *GATA2* deficiency may be lobular or septal. Scleroderma-like changes deep in the subcutis (resembling deep morphoea) have been described [27].

##### Deficiency of Adenosine Deaminase 2 Deficiency

DADA2 is an autosomal recessive disorder caused by compound heterozygous missense mutations in *ADA2.* Skin manifestations may be the presenting symptoms of DADA2. They often include widespread livedo reticularis (often of the racemosa subtype) involving all the limbs and (in some cases) extending to the abdomen or trunk. Other skin manifestations include nodules, EN–like lesions, purpura, leg ulcers, and Raynaud symptoms [28,29]. Any diagnosis of polyarteritis nodosa in a child should always exclude DADA2, since early treatment with an anti-TNF may prevent a stroke. 

###### Histopathology

A biopsy of the skin nodules may have much the same features as polyarteritis nodosa, with fibrinoid necrotising vasculitis affecting the small arteries (in acute panniculitis) and the dermal-subcutaneous junction (at the subacute or reparative stage). Other cases have been characterised by thrombosis of dermal and/or hypodermal capillaries, in the absence of vasculitis [29]. Overall, livedo racemosa with a large branching pattern, nodules or ulceration in a context of neurovascular events, recurrent fever, low immunoglobulin M levels, or paediatric onset is suggestive of DADA2.

### 2.5. Recurrent Lipoatrophic Panniculitis of Children

This term corresponds to a particular clinical presentation of idiopathic lobular panniculitis. It usually starts in childhood and is associated with fever, the systemic involvement of inflamed visceral fat, and (at the end of the inflammatory process) lipoatrophy. Other terms (such as “connective tissue panniculitis”, “lipophagic panniculitis”, and “annular lipoatrophy of the ankles”) have been used to describe cases with similar features. Use of the term “connective tissue panniculitis” is unfortunate because it may lead to confusion with other typical forms of panniculitis associated with connective tissue disorders (e.g., lupus panniculitis or panniculitis of dermatomyositis. Children with recurrent lipoatrophic panniculitis have inflamed, tender, subcutaneous nodules that appear mainly on the limbs (Figure 5A). In some cases, violaceous discoloration of the overlying skin is present. New lesions appear in subsequent attacks, with fever and slight malaise. Older lesions tend to subside and leave a striking, concentric, annular lipoatrophy.

#### Histopathology

Recurrent lipoatrophic panniculitis of children is a lobular form with a mixed but predominantly lymphocytic infiltrate that also contains histiocytes, eosinophils, plasma cells, and neutrophils (Figure 5B,C). The degree of lipoatrophy is variable. Lipidised giant cells are usually seen in the conditions called “lipophagic panniculitis of children” and “annular lipoatrophy of the ankles”. Several cases have been linked to autoimmune disorders: insulin-dependent diabetes mellitus, juvenile rheumatoid arthritis, Graves’ disease, Hashimoto thyroiditis, alopecia areata, vitiligo, coeliac disease, Raynaud’s phenomenon, Crohn’s disease, and partial IgA deficiency [30].

### 2.6. Panniculitis in Self-Healing Juvenile Cutaneous Mucinosis (SHJCM) 

SHJCM typically presents as an acute eruption in an otherwise healthy child. The cutaneous lesions occur suddenly 3 to 10 days after prodromal fever and spread within a few days to become subcutaneous noninflammatory nodules on the head, hands, elbows, arms, and knees (Figure 6A,B). Additional nontender ivory-white papules may arise on the hands, head, and trunk at disease onset or during disease progression. Nodules are a common clinical feature and can precede the papules. Periorbital oedema, asthenia, and joint pain may also be encountered.

#### Histopathology

In the absence of mucinous papules, the diagnosis of SHJCM is challenging. A histopathological assessment of the papules reveals moderate dermal mucin deposition, whereas the nodules show features of proliferative fasciitis or non-specific chronic lobular panniculitis (Figure 6C) [31]. 

## 3. Paediatric Aspects of Forms of Panniculitis That Occur Mainly in Adults

### 3.1. Erythema Nodosum

**Figure 7:**
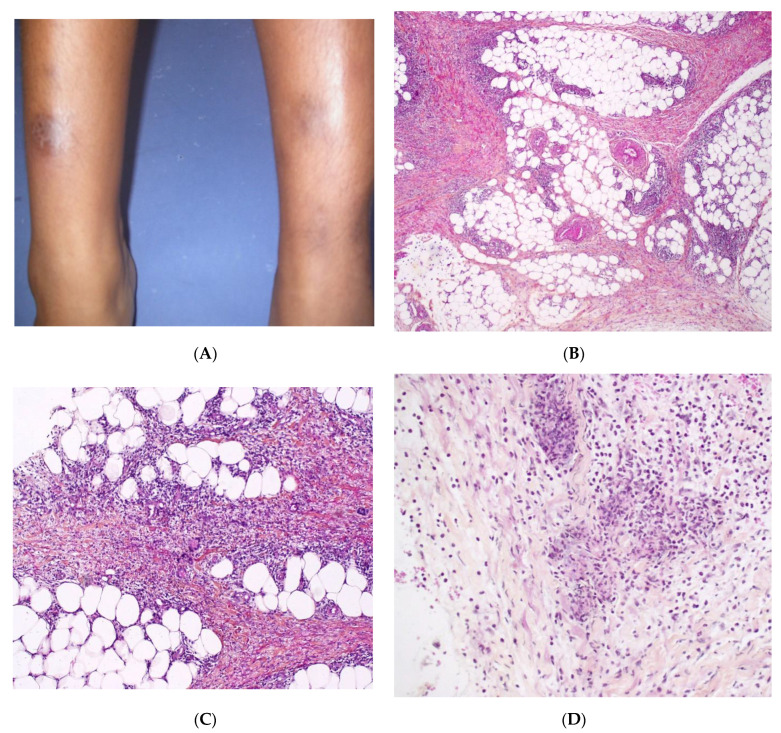
Erythema nodosum. (**A**) Nodules of the legs in an 8-year-old child. *Photo Dermatology Department Hopital Necker Enfants Malades. Paris France*. (**B**) Fibrosing septal pattern with involvement of the periphery of the fat lobules. The process extends into the fat lobules (original magnification ×40). (**C**) Thickened septa of subcutaneous fat with granulomatous inflammation (original magnification ×100). (**D**) Miescher granuloma consisting of small nodular aggregation of histiocytes around a central stellate-shaped cleft located near the junction of the adipocytes and the septum (original magnification ×200).

Although EN is the most common form of panniculitis at all ages, the incidence peaks during adolescence. There is no sex predominance [32]. In children, lesions are most frequent on the anterior aspects of the thighs, upper limbs, trunk, and face (Figure 7A). The aetiology is similar to that of adult EN, with streptococcal and gastrointestinal infections as the main causes. Idiopathic EN accounts for 40% of cases in children. A skin biopsy is not generally performed, but is advisable for patients with an atypical presentation or a protracted course of disease.

#### Histopathology

The aspect is similar to that of EN in adults, with mostly septal panniculitis. The septa of the subcutaneous fat are thickened, and an inflammatory cell infiltrate is present (Figure 7B,C) Neutrophils predominate in acute lesions, whereas mononuclear cells and histiocytes predominate in chronic lesions. Miescher’s radial granuloma—an aggregation of histiocytes around a central stellate or “banana-shaped” cleft—is specific for EN but is not always present (Figure 7D).

### 3.2. Infectious Panniculitis

Infectious panniculitides are very rare and mostly occur in immunosuppressed patients. Several pathogens can infect the subcutaneous fat.

#### Histopathology

Infectious panniculitis is mostly lobular, with a predominance of neutrophils in the infiltrate. Necrosis and suppurative granulomas are frequent but vasculitis is rare. Microorganisms are rarely found in biopsies from non-immunocompromised patients. Bacteriological cultures may be required in immunocompromised patients.

### 3.3. Pancreatic Panniculitis

Pancreatic panniculitis is very rare in children. The aetiology of pancreatitis in children differs from that seen in adults; it arises in a systemic disease setting that often involves a genetic disorder [33,34]. The histopathological features and management are much the same as for the adult disease; lobular panniculitis exhibits mixed inflammation, with coagulative necrosis of adipocytes and ghost cells. Calcification is common.

### 3.4. Panniculitis during Vemurafenib Treatment

Panniculitis has been reported in children treated with vemurafenib for neural tumours of the central nervous system or for Langerhans cell histiocytosis [35,36]. The paediatric cases correspond to the adult phenotype with cutaneous nodules appearing soon after vemurafenib initiation. Vemurafenib panniculitis may be accompanied by fever, which (along with the presence of polymorphonuclear cells in a histological assessment) might prompt the physician to suspect an infectious cause in immunocompromised patients with new skin nodules. The lesions may or may not resolve spontaneously in patients who continue treatment with vemurafenib. 

#### Histopathology

Vemurafenib panniculitis is a mostly lobular neutrophilic form, and lesion cultures for microorganisms are negative.

### 3.5. Factitial and Iatrogenic Panniculitis

Factitial panniculitis (the iatrogenic consequence of certain drug injections) is rare in children. The clinical features of factitial panniculitis are quite variable and depend on the causative agent [37]. In young children, factitial lesions should prompt the clinician to consider a diagnosis of Munchausen syndrome [38]. Subcutaneous aluminium granuloma can develop at the injection site weeks, months, or even years (median: 3 months) after the administration of aluminium-absorbed vaccines (diphtheria/tetanus/pertussis, hepatitis A, hepatitis B, and human papillomavirus vaccines). Factitial panniculitis presents as single or multiple itching subcutaneous nodules and resolves spontaneously.

#### Histopathology

**Figure 8:**
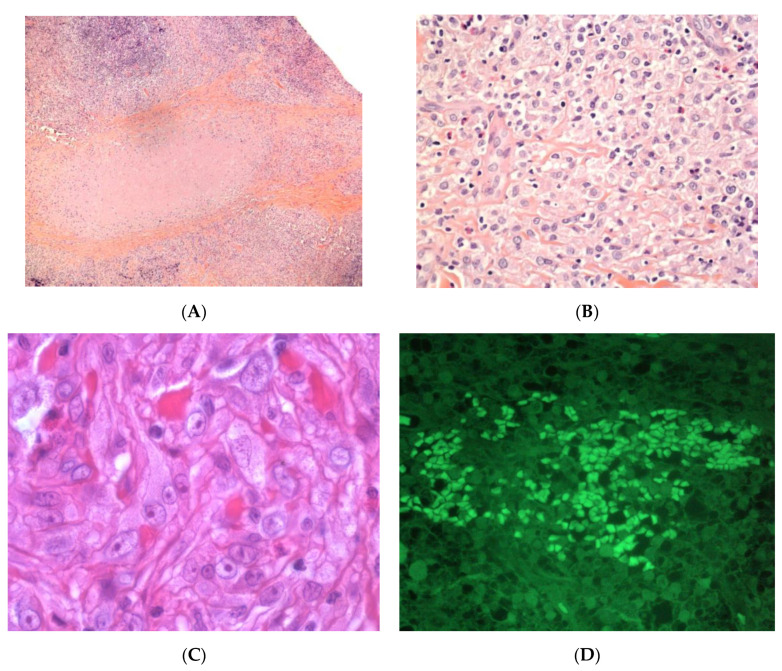
Subcutaneous aluminium granuloma. (**A**) Dense, deep dermal and subcutaneous nodular infiltrate surrounding areas of necrosis (original magnification ×80). (**B**) Mixed infiltrate of lymphocytes, histiocytes, and eosinophils (original magnification ×250). (**C**) Histiocytes containing violaceous granular within their cytoplasm (original magnification ×400). (**D**) The granules stain positive with aluminon staining (Morin, (original magnification ×200)), which reveals the existence of aluminium.

Biopsies are not usually performed in cases of factitial panniculitis but they usually reveal an acute, lobular form of panniculitis associated with fat necrosis and an abundant, predominantly neutrophilic inflammatory infiltrate. Post-vaccination subcutaneous aluminium granuloma shows a dense, deep dermal and subcutaneous nodular mixed infiltrate of lymphocytes, histiocytes, and eosinophils, with germinal centre formation in some cases [39,40]. The bluish, amphophilic granular cytoplasm observed in most of the histiocytes is a characteristic feature of “aluminium granulomas”. Moreover, the granules stain positive for aluminium (Morin reagent) (Figure 8).

### 3.6. Alpha-1-Antitrypsin Deficiency

Panniculitis caused by alpha-1-antitrypsin is a rare autosomal recessive disorder in adults and is even rarer in children. Panniculitis occurs most commonly in individuals who are homozygous for the Z allele of the *SERPINA1* alpha-antitrypsin gene [41]. In most paediatric cases, lesions develop on trauma-damaged areas. Zones of erythema and induration are observed and often mimic cellulitis. The zones rapidly progress to deep nodules with ulceration and exudation of an oily substance produced by adipocyte necrosis. The lesions relapse and heal with atrophic scarring. A paediatric disease onset does not mean that the prognosis is more favourable, because lesions can continue to appear into adulthood. The panniculitis may precede (sometimes by several years) emphysema and other manifestations of an antitrypsin deficiency or may be the only clinical sign [42].

#### Histopathology

The combination of extensive liquefactive necrosis of the dermis, neutrophilic inflammation, and destructive changes in the fibrous septa of the subcutis (resulting in separation of the subcutis’ lobules) is pathognomonic for alpha 1-antitrypsin deficiency [43]. 

### 3.7. H Syndrome (OMIM #612391)

H syndrome (OMIM #612391) is a recently described autosomal recessive genodermatosis, the systemic manifestations of which are linked to mutations in the *SLC29A3* gene (coding for a nucleoside transporter). The disease is characterised by the progressive, sclerodermatous thickening of the skin with overlying hyperpigmentation and hypertrichosis located mainly on the upper inner thighs. The involvement of the genitalia, lower trunk, and limbs is variable. The additional systemic manifestations that gave the syndrome its “H” name included hepatosplenomegaly, hearing loss, heart anomalies, hypogonadism, low height, hallux valgus with fixed flexion contractures of the toe joints and proximal interphalangeal joints of the hands, and (occasionally) hyperglycaemia/diabetes mellitus. Induration of the skin is often observed.

#### Histopathology

The dermis and hypodermis contain interstitial chronic inflammatory infiltrates mainly consisting of monocyte-derived cells (small CD68+, large CD68+, CD163+, S100+, CD1a- histiocytes with emperipolesis, and CD34+ and FXIIIa+ dendrocytes) and plasma cells. This inflammatory component is associated with striking fibrosis of the subcutis and moderate fibrosis of the dermis [44]. 

### 3.8. Panniculitis Associated with Connective Tissue Diseases and Vasculitis

#### 3.8.1. Lupus Erythematosus Panniculitis (LEP)

Lupus erythematosus panniculitis is very rare in children and is often found as an isolated phenomenon with no systemic or other cutaneous findings. However, LEP can be observed in patients with discoid lupus erythematosus [45], systemic lupus erythematosus [46], or (very rarely) neonatal lupus [47].

Clinically, LEP manifests itself as tender nodules or plaques, with female predominance. In children, the most common lesion sites are the face, upper arms, and shoulders [48]. The lesions often heal with scarring and lipoatrophy.

##### Histopathology

**Figure 9:**
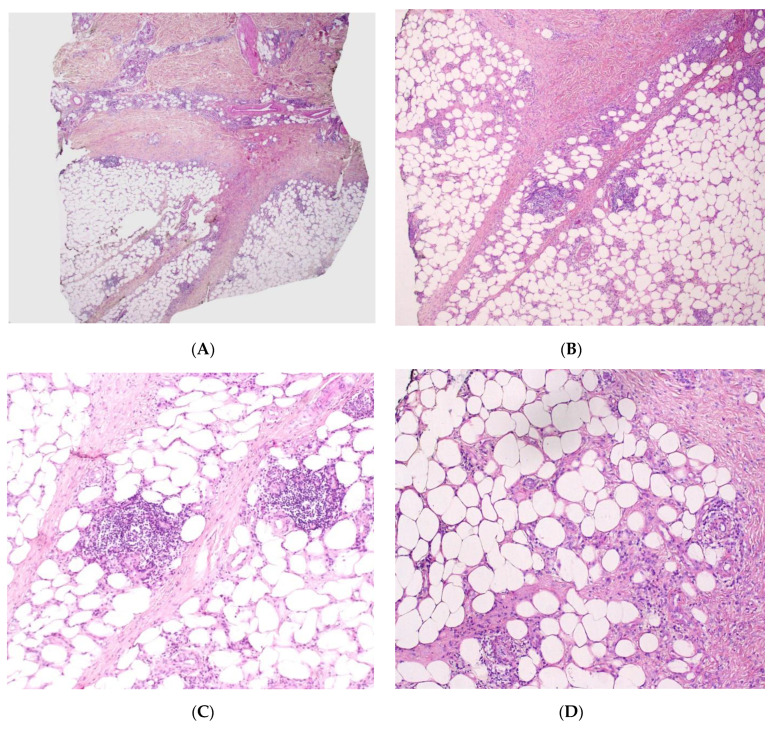
Lupus panniculitis. (**A**) Dense dermal infiltrate with extension into subcutaneous fat (original magnification ×30). (**B**) Septae appear fibrotic (original magnification ×100). (**C**) Lymphoid follicles (original magnification ×120). (**D**) Necrosis of adipocytes with hyalinisation of the fat lobular panniculitis with lymphocytes among necrotic adipocytes (original magnification ×120).

LEP is a lobular panniculitis with a prominent lymphocytic infiltrate (lymphocytic lobular panniculitis) (Figure 9). Concomitant septal involvement is often present, with sclerosing and thickening of the septa. Myxoid and hyaline changes may be found in the connective tissue septa and the lower dermis. Lymphocytic vasculitis with lymphocytic nuclear dust is sometimes present. A characteristic feature includes fat necrosis and the presence of lymphoid follicles (sometimes with germinal centres) adjacent to the fibrous septa. Plasma cells are present in many cases. Epidermal and dermal changes (particularly basal vacuolar changes) may be present [49].

#### 3.8.2. Panniculitis and Dermatomyositis (DM)

Panniculitis is a rare clinical finding in juvenile DM and may be the disease’s only cutaneous manifestation. The typical clinical picture involves erythematous, painful subcutaneous nodules or plaques that typically occur on the buttocks, thighs, and arms and are often attributed to a flare of DM [50]. Migratory subcutaneous nodules have also been described. 

##### Histopathology

Panniculitis in DM is very similar to lupus panniculitis. It is mostly lobular, with lymphocytes and plasma cells found among the adipocytes. The collagen bundles of the septa show hyaline sclerosis, and the fat is progressively replaced by fibrous tissue [50]. In DM, panniculitis associated with calcification of muscle and deep tissue is more common than isolated panniculitis. In cases with calcification, the fat lobules show lipophagic granuloma and various degrees of acute and/or chronic inflammation [50].

### 3.9. Cytotoxic T-Cell Clonal Panniculitis

Subcutaneous panniculitis-like cytotoxic T-cell clonal proliferation arises mainly in adults and is very rare in children. The prognosis in children is usually excellent, and thus, the term “cytotoxic T-cell clonal panniculitis” is more appropriate than “subcutaneous panniculitis-like cytotoxic T-cell lymphoma” [51]. This T-cell clonal proliferation is frequently associated with an autoimmune disease, and thus, a thorough diagnostic work-up is required. The condition can appear after an infection like toxoplasmosis, chicken pox, and viral nasopharyngitis. Cytotoxic T-cell clonal panniculitis might result from a dysregulation of T cell responses after recognition of an autoantigen or an antigen from a pathogen [52]. Cases with a severe haemophagocytic syndrome are significantly linked with germline mutations in *HAVCR2* gene encoding T-cell immunoglobulin mucin-3 (TIM-3), an inhibitory receptor expressed on T-cells and innate immune cells. *HAVCR2* mutations are found in 60–85% of cases of cytotoxic T-cell clonal panniculitis and are associated with younger age at onset and a more frequent association with haemophagocytic lymphohistiocytosis [53]. Detection of a TIM-3 deficiency provides a rationale for treatment with anti-inflammatory agent (steroids or interferon) rather than polychemotherapy.

Clinically, patients present with solitary or multiple nodules and erythematous subcutaneous plaques, which are predominantly located on the limbs and trunk. The lesions have overlying erythema, are usually painless, and range in size from 0.5–2.0 cm. Lesions at different stages of healing may be observed, which suggests a relapsing-remitting clinical course. Lipoatrophy or persistent facial swelling may be associated with these cutaneous manifestations. A haemophagocytic syndrome is sometimes also present, albeit far less frequently in children than in adults. The syndrome’s features including fever, chills, malaise, weight loss, pancytopenia, lymphadenopathy, hepatosplenomegaly, abnormal liver enzyme levels, and coagulation disorders [54].

#### Histopathology

**Figure 10:**
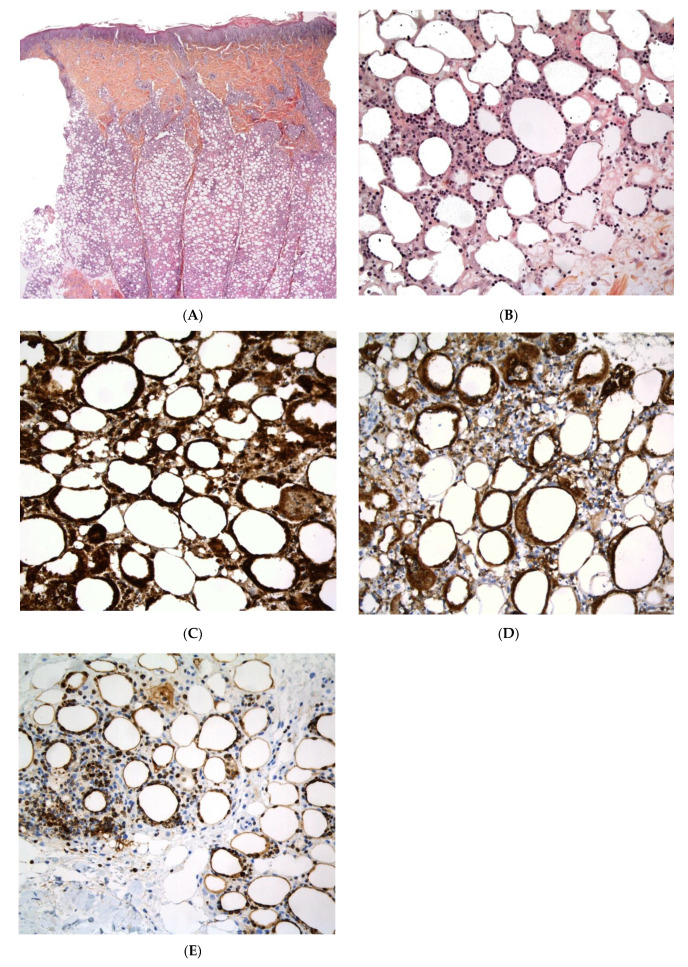
Cytotoxic T clonal panniculitis. (**A**) Lobular panniculitis with lymphoid cell infiltration (original magnification ×30). (**B**) Atypical lymphoid cells with adipocyte rimming and adipocyte necrosis showing infiltration by cells expressing (**C**) CD3, (**D**) Tia1, and (**E**) granzyme (original magnification ×400).

Cytotoxic T-cell clonal panniculitis is a lobular panniculitis with a dense, nodular or diffuse infiltrate of small to medium pleomorphic lymphocytes in the subcutaneous fat (Figure 10). The cells are arranged around the adipocytes—the so-called “rimming of the adipocytes”. Necrosis is often a prominent feature and may mask specific histopathological features. The pathologist can search for small aggregates of atypical lymphocytes within the necrotic region, together with ghost cells (necrotic lymphocytes). Apoptosis with karyorrhectic debris is frequently associated with the presence of haemophagocytic cells. Paediatric lesions often contain plasma cells. In some cases, the specific features are confined to a small portion of the subcutaneous fat; a thorough examination of the biopsy specimen and multiple serial sections of the skin biopsy are needed for a firm diagnosis. Immunohistochemical studies show that the lymphocytes are mostly cytotoxic CD8+ cells expressing the cytotoxic proteins TIA1, granzyme B, and perforin, but not CD56. In situ hybridisation (to detect the EBER-1 small RNA from the Epstein-Barr virus) is usually negative [55]. 

T-cell clonality can be demonstrated by identifying αβT-cell receptor rearrangements using polymerase chain reactions or high-throughput sequencing. 

In most cases, the prognosis is excellent, and conservative treatment with an immunosuppressant is recommended [56]. Some cases may even regress spontaneously. In patients with an immunodeficiency (particularly TIM3 deficiency), bone marrow transplantation is curative. Patients with cytotoxic T-cell clonal panniculitis and haemophagocytic syndrome should always be screened for an immunodeficiency.

The term “cytophagic histiocytic panniculitis” has been used to describe subcutaneous nodules or plaques that show (in a histological examination) macrophage infiltrates also containing erythrocytes, lymphocytes, and/or karyorrhectic debris (i.e., haematophagocytosis). With the advent of immunophenotyping and genetic techniques, it has become apparent that the vast majority of patients have lymphoma, with atypical lymphoid cells also present in the panniculus; hence, the term “cytophagic histiocytic panniculitis” is no longer used.

## 4. Conclusions

Panniculitis in children is rare but can have many possible causes. Certain types are specific for childhood, whereas others are rare at a young age. A biopsy is not always necessary but is often useful if sufficient tissue is available; clinicians must, therefore, choose the best time and best site for sampling. Patients with panniculitis but who lack an aetiological diagnosis should be monitored for immunodeficiency and autoinflammatory diseases.

## Figures and Tables

**Table 1:** The histopathologic classification of lobular panniculitis in children.

**No Vasculitis**	
*When lymphocytes are predominant:*	
With superficial and deep perivascular dermal infiltrate	Cold panniculitis
With lymphoid follicles, plasma cells, and lymphocyte nuclear dust	Lupus panniculitis
With lymphocytes and plasma cells	Dermatomyositis
	TRAPS
With small to medium-sized pleomorphic lymphocytes	Cytotoxic T-cell panniculitis
With histiocytes, eosinophils, plasma cells, and neutrophils	Recurrent lipoatrophic panniculitis of children along with variable degrees of lipoatrophy
*When neutrophils are predominant:*	
With extensive fat necrosis and saponification of adipocytes	Pancreatic panniculitis
With neutrophils between collagen bundles in the deep reticular dermis	Alpha1-antitrypsin deficiency
With bacteria, fungi, or protozoa identified by specific stains	Infective panniculitis
With foreign bodies	Factitial panniculitis
	Panniculitis during vemurafenib treatment
	Familial Mediterranean fever
*When mononuclear atypical cells are predominant*	
With neutrophils	CANDLE syndrome
*When histiocytes are predominant*	
Histiocytes with enlarged, amphophilic cytoplasm	Post-vaccination subcutaneous aluminium granuloma
No crystals in adipocytes	Blau syndrome
With crystals in histiocytes or adipocytes	SFN, post-steroid panniculitis
With interstitial monocyte-derived cells and fibrosis in the dermis and hypodermis	H syndrome.
**With Vasculitis **	
	Deficiency in ADA2
	Otulopenia

## References

[1] Bodemer C. (2019). Panniculitis in Harper’s Textbook of Pediatric Dermatology.

[2] Torrelo A., Hernández A. (2008). Panniculitis in children. Dermatol. Clin..

[3] Polcari I.C., Stein S.L. (2010). Panniculitis in childhood. Dermatol. Ther..

[4] Requena L., Yus E.S. (2001). Panniculitis. Part I. Mostly septal panniculitis. J. Am. Acad. Dermatol..

[5] Requena L., Yus E.S. (2001). Panniculitis. Part II. Mostly lobular panniculitis. J. Am. Acad. Dermatol..

[6] Del Pozzo-Magaña B.R., Ho N. (2016). Subcutaneous Fat Necrosis of the Newborn: A 20-Year Retrospective Study. Pediatr. Dermatol..

[7] Lara L.G., Villa A.V., Rivas M.M., Capella M.S., Prada F., Enseñat M.A.G. (2017). Subcutaneous Fat Necrosis of the Newborn: Report of Five Cases. Pediatr. Neonatol..

[8] Stefanko N.S., Drolet B.A. (2019). Subcutaneous fat necrosis of the newborn and associated hypercalcemia: A systematic review of the literature. Pediatr. Dermatol..

[9] Ricardo-Gonzalez R.R., Lin J.R., Mathes E.F., McCalmont T.H., Pincus L.B. (2016). Neutrophil-rich subcutaneous fat necrosis of the newborn: A potential mimic of infection. J. Am. Acad. Dermatol..

[10] Zeb A., Darmstadt G.L. (2008). Sclerema neonatorum: A review of nomenclature, clinical presentation, histological features, differential diagnoses and management. J. Perinatol..

[11] Silverman R.A., Newman A.J., LeVine M.J., Kaplan B. (1988). Poststeroid panniculitis: A case report. Pediatr. Dermatol..

[12] Malia L., Wang A., Scheiner L., Laurich V.M. (2019). Cold Panniculitis After Ice Therapy for Supraventricular Tachycardia. Pediatr. Emerg. Care.

[13] Bader-Meunier B., Rieux-Laucat F., Touzot F., Frémond M.L., André-Schmutz I., Fraitag S., Bodemer C. (2018). Inherited Immunodeficiency: A New Association with Early-Onset Childhood Panniculitis. Pediatrics.

[14] Escudier A., Mauvais F.X., Bastard P., Boussard C., Jaoui A., Koskas V., Lecoq E., Michel A., Orcel M.C., Truelle P.E. (2018). Peau et fièvres récurrentes auto-inflammatoires [Dermatological features of auto-inflammatory recurrent fevers]. Arch. Pediatr..

[15] Torrelo A., Patel S., Colmenero I., Gurbindo D., Lendínez F., Hernández A., López-Robledillo J.C., Dadban A., Requena L., Paller A.S. (2010). Chronic atypical neutrophilic dermatosis with lipodystrophy and elevated temperature (CANDLE) syndrome. J. Am. Acad. Dermatol..

[16] Ohmura K. (2019). Nakajo-Nishimura syndrome and related proteasome-associated autoinflammatory syndromes. J. Inflamm. Res..

[17] Volpi S., Picco P., Caorsi R., Candotti F., Gattorno M. (2016). Type I interferonopathies in pediatric rheumatology. Pediatr. Rheumatol. Online J..

[18] Torrelo A., Colmenero I., Requena L., Paller A.S., Ramot Y., Richard Lee C.C., Vera A., Zlotogorski A., Goldbach-Mansky R., Kutzner H. (2015). Histologic and Immunohistochemical Features of the Skin Lesions in CANDLE Syndrome. Am. J. Dermatopathol..

[19] Figueras-Nart I., Mascaró J.M., Solanich X., Hernández-Rodríguez J. (2019). Dermatologic and Dermatopathologic Features of Monogenic Autoinflammatory Diseases. Front. Immunol..

[20] Leiva-Salinas M., Betlloch I., Arribas M.P., Francés L., Pascual J.C. (2014). Neutrophilic lobular panniculitis as an expression of a widened spectrum of familial mediterranean fever. JAMA Dermatol..

[21] Zhou Q., Yu X., Demirkaya E., Deuitch N., Stone D., Tsai W.L., Kuehn H.S., Wang H., Yang D., Park Y.H. (2016). Biallelic hypomorphic mutations in a linear deubiquitinase define otulipenia, an early-onset autoinflammatory disease. Proc. Natl. Acad. Sci. USA.

[22] Poline J., Fogel O., Pajot C., Miceli-Richard C., Rybojad M., Galeotti C., Grouteau E., Hachulla E., Brissaud P., Cantagrel A. (2020). Early-onset granulomatous arthritis, uveitis and skin rash: Characterization of skin involvement in Blau syndrome. J. Eur. Acad. Dermatol. Venereol..

[23] Wouters C.H., Martin T.M., Stichweh D., Punaro M., Doyle T.M., Lewis J.A., Quartier P., Rose C.D. (2007). Infantile onset panniculitis with uveitis and systemic granulomatosis: A new clinicopathologic entity. J. Pediatr..

[24] Toro J.R., Aksentijevich I., Hull K., Dean J., Kastner D.L. (2000). Tumor necrosis factor receptor-associated periodic syndrome: A novel syndrome with cutaneous manifestations. Arch. Dermatol..

[25] Cattalini M., Meini A., Monari P., Gualdi G., Arisi M., Pelucchi F., Bolognini S., Gattorno M., Calzavara-Pinton P.G., Plebani A. (2013). Recurrent migratory angioedema as cutaneous manifestation in a familiar case of TRAPS: Dramatic response to Anakinra. Dermatol. Online J..

[26] Polat A., Dinulescu M., Fraitag S., Nimubona S., Toutain F., Jouneau S., Poullot E., Droitcourt C., Dupuy A. (2018). Skin manifestations among GATA2-deficient patients. Br. J. Dermatol..

[27] Spinner M.A., Sanchez L.A., Hsu A.P., Shaw P.A., Zerbe C.S., Calvo K.R., Arthur D.C., Gu W., Gould C.M., Brewer C.C. (2014). GATA2 deficiency: A protean disorder of hematopoiesis, lymphatics, and immunity. Blood.

[28] Chasset F., Fayand A., Moguelet P., Kouby F., Bonhomme A., Franck N., Goldman-Lévy G., Fraitag S., Barbaud A., Queyrel V. (2020). Clinical and pathological dermatological features of deficiency of adenosine deaminase 2: A multicenter, retrospective, observational study. J. Am. Acad. Dermatol..

[29] Shwin K.W., Lee C.R., Goldbach-Mansky R. (2017). Dermatologic Manifestations of Monogenic Autoinflammatory Diseases. Dermatol. Clin..

[30] Torrelo A., Noguera-Morel L., Hernández-Martín A., Clemente D., Barja J.M., Buzón L., Azorín D., de Jesús A.A., López-Robledillo J.C., Colmenero I. (2017). Recurrent lipoatrophic panniculitis of children. J. Eur. Acad. Dermatol. Venereol..

[31] Luchsinger I., Coulombe J., Rongioletti F., Haspeslagh M., Dompmartin A., Melki I., Dagher R., Bader-Meunier B., Fraitag S., Bodemer C. (2018). Self-healing juvenile cutaneous mucinosis: Clinical and histopathologic findings of 9 patients: The relevance of long-term follow-up. J. Am. Acad. Dermatol..

[32] Moraes A.J., Soares P.M., Zapata A.L., Lotito A.P., Sallum A.M., Silva C.A. (2006). Panniculitis in childhood and adolescence. Pediatr. Int..

[33] Boyd A.S., Wester A.C. (2019). Pancreatic fat necrosis in a 10-year-old girl: A case report and review of the literature. Pediatr. Dermatol..

[34] Holstein T., Horneff G., Wawer A., Gaber G., Burdach S. (2000). Panniculitis, pancreatitis and very severe aplastic anemia in childhood: A challenge to treat. Ann. Hematol..

[35] Boull C.L., Gardeen S., Abdali T., Li E., Potts J., Rubin N., Carlberg V.M., Gupta D., Hunt R., Luu M. (2020). Cutaneous Reactions in Children Treated with MEK Inhibitors, BRAF Inhibitors, or Combination Therapy: A Multi-Center Study. J. Am. Acad. Dermatol..

[36] Finelt N., Lulla R.R., Melin-Aldana H., Ruth J.S., Lin F.Y., Su J.M., Pourciau C.Y., Hunt R.D., Kenner-Bell B.M. (2017). Bumps in the Road: Panniculitis in Children and Adolescents Treated with Vemurafenib. Pediatr. Dermatol..

[37] Sanmartín O., Requena C., Requena L. (2008). Factitial panniculitis. Dermatol. Clin..

[38] Boyd A.S., Ritchie C., Likhari S. (2014). Munchausen syndrome and Munchausen syndrome by proxy in dermatology. J. Am. Acad. Dermatol..

[39] Chong H., Brady K., Metze D., Calonje E. (2006). Persistent nodules at injection sites (aluminium granuloma)—Clinicopathological study of 14 cases with a diverse range of histological reaction patterns. Histopathology.

[40] Haag C.K., Dacey E., Hamilton N., White K.P. (2019). Aluminum granuloma in a child secondary to DTaP-IPV vaccination: A case report. Pediatr. Dermatol..

[41] Torres-Durán M., Lopez-Campos J.L., Barrecheguren M., Miravitlles M., Martinez-Delgado B., Castillo S., Escribano A., Baloira A., Navarro-Garcia M.M., Pellicer D. (2018). Alpha-1 antitrypsin deficiency: Outstanding questions and future directions. Orphanet J. Rare Dis..

[42] Hendrick S.J., Silverman A.K., Solomon A.R., Headington J.T. (1988). Alpha 1-antitrypsin deficiency associated with panniculitis. J. Am. Acad. Dermatol..

[43] Blanco I., Lipsker D., Lara B., Janciauskiene S. (2016). Neutrophilic panniculitis associated with alpha-1-antitrypsin deficiency: An update. Br. J. Dermatol..

[44] Doviner V., Maly A., Ne’eman Z., Qawasmi R., Aamar S., Sultan M., Spiegel M., Molho-Pessach V., Zlotogorski A. (2010). H syndrome: Recently defined genodermatosis with distinct histologic features. A morphological, histochemical, immunohistochemical, and ultrastructural study of 10 cases. Am. J. Dermatopathol..

[45] Wimmershoff M.B., Hohenleutner U., Landthaler M. (2003). Discoid lupus erythematosus and lupus profundus in childhood: A report of two cases. Pediatr. Dermatol..

[46] Zhang R., Dang X., Shuai L., He Q., He X., Yi Z. (2018). Lupus erythematosus panniculitis in a 10-year-old female child with severe systemic lupus erythematosus: A case report. Medicine.

[47] Nitta Y. (1997). Lupus erythematosus profundus associated with neonatal lupus erythematosus. Br. J. Dermatol..

[48] Weingartner J.S., Zedek D.C., Burkhart C.N., Morrell D.S. (2012). Lupus erythematosus panniculitis in children: Report of three cases and review of previously reported cases. Pediatr. Dermatol..

[49] Park H.S., Choi J.W., Kim B.K., Cho K.H. (2010). Lupus erythematosus panniculitis: Clinicopathological, immunophenotypic, and molecular studies. Am. J. Dermatopathol..

[50] Neidenbach P.J., Sahn E.E., Helton J. (1995). Panniculitis in juvenile dermatomyositis. J. Am. Acad. Dermatol..

[51] Bader-Meunier B., Fraitag S., Janssen C., Brochard K., Lamant L., Wouters C., Bodemer C. (2013). Clonal cytophagic histiocytic panniculitis in children may be cured by cyclosporine A. Pediatrics.

[52] Michonneau D., Petrella T., Ortonne N., Ingen-Housz-Oro S., Franck N., Barete S., Battistella M., Beylot-Barry M., Vergier B., Maynadié M. (2017). Subcutaneous Panniculitis-like T-cell Lymphoma: Immunosuppressive Drugs Induce Better Response than Polychemotherapy. Acta. Derm. Venereol..

[53] Gayden T., Sepulveda F.E., Khuong-Quang D.A., Pratt J., Valera E.T., Garrigue A., Kelso S., Sicheri F., Mikael L.G., Hamel N. (2018). Germline HAVCR2 mutations altering TIM-3 characterize subcutaneous panniculitis-like T cell lymphomas with hemophagocytic lymphohistiocytic syndrome. Nat. Genet..

[54] Chaweephisal P., Sosothikul D., Polprasert C., Wananukul S., Seksarn P. (2021). Subcutaneous Panniculitis-like T-Cell Lymphoma With Hemophagocytic Lymphohistiocytosis Syndrome in Children and Its Essential Role of HAVCR2 Gene Mutation Analysis. J. Pediatr. Hematol. Oncol..

[55] Rutnin S., Porntharukcharoen S., Boonsakan P. (2019). Clinicopathologic, immunophenotypic, and molecular analysis of subcutaneous panniculitis-like T-cell lymphoma: A retrospective study in a tertiary care center. J. Cutan. Pathol..

[56] Singh S., Philip C.C., John M.J. (2019). Pediatric Subcutaneous Panniculitis-like T-Cell Lymphoma with Hemophagocytosis Showing Complete Resolution with the BFM90 Protocol: Case Report and Review of Literature. J. Pediatr. Hematol. Oncol..

